# Janus Monolayer of 1T-TaSSe: A Computational Study

**DOI:** 10.3390/ma17184591

**Published:** 2024-09-19

**Authors:** Karol Szałowski

**Affiliations:** Department of Solid State Physics, Faculty of Physics and Applied Informatics, University of Lodz, Ulica Pomorska 149/153, 90-236 Lodz, Poland; e-karol.szalowski@uni.lodz.pl

**Keywords:** charge density wave, periodic lattice deformation, Janus monolayer, transition metal dichalcogenide, monolayer, tantalum disulfide, tantalum diselenide

## Abstract

Materials exhibiting charge density waves are attracting increasing attention owing to their complex physics and potential for applications. In this paper, we present a computational, first principles-based study of the Janus monolayer of 1T-TaSSe transition metal dichalcogenide. We extensively compare the results with those obtained for parent compounds, TaS_2_ and TaSe_2_ monolayers, with confirmed presence of $\sqrt{13}\times \sqrt{13}$ charge density waves. The structural and electronic properties of the normal (undistorted) phase and distorted phase with $\sqrt{13}\times \sqrt{13}$ periodic lattice distortion are discussed. In particular, for a normal phase, the emergence of dipolar moment due to symmetry breaking is demonstrated, and its sensitivity to an external electric field perpendicular to the monolayer is investigated. Moreover, the appearance of imaginary energy phonon modes suggesting structural instability is shown. For the distorted phase, we predict the presence of a flat, weakly dispersive band related to the appearance of charge density waves, similar to the one observed in parent compounds. The results suggest a novel platform for studying charge density waves in two-dimensional transition metal dichalcogenides.

## 1. Introduction

Janus monolayers are novel two-dimensional materials with different properties in the top and bottom layers of the structure, which is usually due to different atoms constituting the top and bottom planes [1] (but also due to the nonequivalent functionalization of both planes, like in Ref. [2]). This two-faced nature of such structures gives rise to a plethora of interesting properties, emerging due to in-built symmetry breaking [3]. A highly interesting, prototypical class of Janus materials is a group of monolayer transition metal dichalcogenides (TMDs) [4,5,6,7], suggested theoretically in 2013 [8], significantly enriching the already intriguing field of two-dimensional TMDs [9,10]. The successful synthesis of MoSSe [4,11] paved the way for remarkable progress in this area of research and inspired further development of various effective synthesis strategies [5]. As a consequence, the subsequent synthesis of such structures as WSSe [12,13], SPtSe [14], MoSH [15], BiTeI [16], and CrTeSe [17] can be noticed. On the other hand, computational studies of a rather wide class of Janus materials have been conducted and reported [18,19,20,21,22].

Selected bulk TMDs are known to host charge density wave (CDW) ordering [23,24,25], which is closely related to the emergence of periodic lattice deformation (PLD), leading to a variety of superstructures. Among them, the 1T polymorph (with transition metal atoms octahedrally coordinated with chalcogen atoms) is best known to form $\sqrt{13}\times \sqrt{13}$-type PLD with a characteristic distortion pattern of transition metal atoms known as a star-of-David cluster, especially characteristic of TaS_2_ and TaSe_2_ [23,24,26,27]. Within this flourishing scope of research, recent achievements in the synthesis of two-dimensional TMDs have focused the interest on monolayer TMD systems with CDWs.

The exploration of CDWs in monolayer materials relies on concerted theoretical and experimental works. Theoretical studies on CDWs with $\sqrt{13}\times \sqrt{13}$ reconstruction in monolayer TMDs involved TaS_2_ [28,29,30,31,32,33,34], TaSe_2_ [33,35,36], TaTe_2_ [33], NbSe_2_ [37,38,39,40], NbS_2_ [41], or NbO_2_ [42]. Such ordering has been verified experimentally in monolayers of TaS_2_ [13,34,43,44,45], TaSe_2_ [13,36,46,47], and NbSe_2_ [46,48]. In relation to Janus materials, recent studies based on density functional theory (DFT) calculations have suggested the presence of various kinds of CDWs in Janus monolayers as well, such as VTeSe [49], MoSH [50], WSH [51,52], WSeH [51], CrSH, TcSH and ReSH [52], TiSeS [53], and CrTeSe [17]. Such predictions stimulate further research focused on CDWs in Janus materials. Moreover, the presence of CDWs in CrTeSe has been verified experimentally [17].

The experimental studies of CDWs have also involved bulk TMD alloys, especially 1T-Ta(S,Se). For example, for 1T-TaS_2−x_Se_x_ bulk samples, the presence of $\sqrt{13}\times \sqrt{13}$ PLD was preserved [54,55]. 1T-TaS_2−x_Se_x_ has also revealed the possibility of switching between various CDW states by laser pulses [56] or the existence of superconductivity [57]. The transport properties of this compound were investigated experimentally [58], and various CDW states in Ta(S,Se) were studied in [59]. The physics of metal-to-insulator transition in 1T-TaS_2−x_Se_x_ is discussed in [60]. Moreover, STM studies of the Ta(S,Se) alloy can be mentioned [61]. In addition, the 1T-TaSe_2−x_Te_x_ alloy has also been studied [62]. This indicates a significant interest in the physics of CDW-forming alloys based on S and Se.

Materials with CDWs are attracting growing attention due to their complex physics and possible applications [49,63,64,65], such as oscillator circuits [66], field effect transistors [67], light sensors [68], fast GHz switches [69], THz modulators [70], nanophotonics devices [71], and memristive elements [72]. Let us also mention that a CDW degree of freedom was predicted and experimentally demonstrated to be useful for controlling the proximity-induced spin–orbit coupling [73,74] in TMD–graphene heterostructures, which constitute particularly promising spintronic platforms [75]. The high potential for applications and rich physics of CDW materials motivate the search for novel systems from this class.

In order to explore the physics of CDW in a Janus structure, it seems interesting to undertake a study of the Janus monolayer TMD based on CDW-forming parent compounds from this range. Let us mention that in a theoretical study of a wide range of TMD Janus structures [19], the TaSSe compound was briefly mentioned among the others, and both TaS_2_ and TaSe_2_ are well known to exhibit CDW ordering in a monolayer form [13,34,36,43,44,45,46,47].

Therefore, motivated by the growing interest in monolayer materials with CDWs, we present in this paper a computational study of the properties of a 1T-TaSSe monolayer Janus TMD based on first principles, DFT-based calculations. The structural properties and band structure are discussed and compared with the corresponding results obtained for parent compounds TaS_2_ and TaSe_2_. Moreover, the influence of the perpendicular electric field on the properties of TaSSe is investigated. Finally, the possibility of formation of commensurate CDWs stemming from a $\sqrt{13}\times \sqrt{13}$ periodic lattice deformation is predicted. Compared to the existing literature of DFT studies of Janus systems, like [22], presenting an application-oriented review of chromium-based materials, our paper only focuses on a single system with a possible transition between the normal phase (without CDW) and the phase with a CDW following structural reconstruction. Both parent compounds of the studied system are known to be synthesized and exhibit the described type of behavior.

The paper is structured as follows. The Methods section describes the computational approach to modeling the structures of interest. The next part, Results and Discussion, is focused on the presentation and analysis of results concerning the structural and electronic properties of TaSSe, both in the normal and CDW phases. Finally, the Conclusions section is focused on the importance of the work, and future prospects are stated.

## 2. Computational Methods

This study is based on first-principles calculations based on DFT [76,77]. All the first-principles calculations were carried out using Quantum ESPRESSO v. 7.0 suite [78,79], implementing DFT with a plane wave basis. In addition, calculations of phonon dispersion relations were carried out using density functional perturbation theory.

In order to simulate monolayer systems of 1T-TaSSe and its parent compounds, TaS_2_ and TaSe_2_, we used a slab geometry with cell dimension perpendicular to the layer equal to 30 Å in order to separate the system from its periodic images (giving the vacuum of at least 25 Å). The calculations for a normal (undistorted) phase were performed in a 1 × 1 cell with 3 atoms, whereas for a distorted (CDW) phase, we used a $\sqrt{13}\times \sqrt{13}$ supercell with 39 atoms. In the calculations, we used scalar relativistic pseudopotentials (for the lattice constant relaxations, the phonon calculations, and the total energy calculations with Fermi–Dirac smearing) and fully relativistic pseudopotentials (in the other cases) [80] with the projector-augmented wave method [81] and the Perdew–Burke–Ernzerhof exchange correlation functional [82]. The cut-off energies for charge density and wavefunctions were equal to 402 Ry and 53 Ry, respectively. For most of the calculations, Methfessel–Paxton smearing of 1 mRy was used for the Brillouin zone integration in self-consistent calculations [83], whereas for the non-self-consistent calculations, a tetrahedron method [84] was used for this purpose. The calculations of the phonon dispersion relations followed the scheme from Ref. [85] for two-dimensional systems. The van der Waals long-range interactions were accounted for in a semiempirical approach [86,87]. For Janus structures, a dipole correction was applied [88] with a piecewise linear potential in the direction perpendicular to the monolayer, permitting the simulation of the external electric field. For a 1 × 1 cell, the lattice constant relaxation and the relaxation of atomic positions was carried out using a 36 × 36 × 1 grid of *k*-points, with convergence thresholds for the total energy equal to $10^{-6}$ Ry and for the total force equal to $10^{-6}$ Ry/$a_{0}$, respectively. The final self-consistent calculation was carried out with a 72 × 72 × 1 grid. The relaxation relied on the Broyden–Fletcher–Goldfarb–Shanno quasi-Newton algorithm. For a $\sqrt{13}\times \sqrt{13}$ cell, a 9 × 9 × 1 mesh was used for the atomic position relaxation and the further calculations, with the convergence thresholds for the total energy equal to $10^{-4}$ Ry and for the total force equal to $10^{-3}$ Ry/$a_{0}$, respectively. For the phononic calculations, a 36 × 36 × 1 grid of *k*-points was used for the electrons and a Monkhorst-pack grid of 8 × 8 × 1 *q*-points served the calculations of the phononic frequencies.

Additionally, the Bader charge analysis was carried out using Bader code [89,90,91]. The visualization of the structures was performed with VESTA software ver. 3.5.7 [92].

## 3. Results and Discussion

In this section, we present and discuss the results of our ab initio modelling of TaSSe, providing an extensive comparison with its parent compounds, TaS_2_ and TaSe_2_. The properties of a normal (undistorted) phase and of a PLD (distorted) phase exhibiting CDW are considered separately.

### 3.1. Normal Phase

Let us start the discussion of the system in question from the undistorted monolayer structure of a 1T polymorph, modelled using a 1 × 1 cell in the calculations, which we call a normal phase.

**Figure 1:**
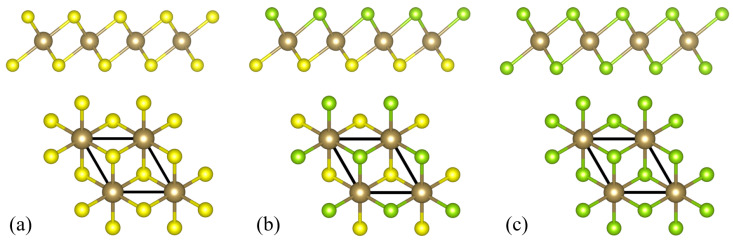
The side and top views of the crystalline structure of a normal phase of 1T polymorph of (**a**) TaS_2_, (**b**) TaSSe, and (**c**) TaSe_2_ monolayer. The unit cell is marked with a thick solid line in the top view. The Ta atoms are marked with brown spheres, S with yellow spheres, and Se with green spheres.

**Figure 2:**
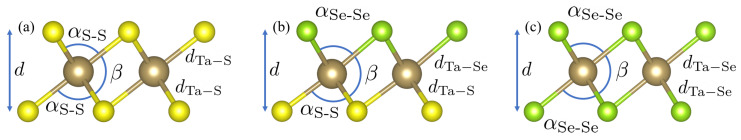
The schematic explanation of the parameters characterizing the crystalline structure of a normal phase of 1T polymorph of (**a**) TaS_2_, (**b**) TaSSe, and (**c**) TaSe_2_ monolayer. The Ta atoms are marked with brown spheres, S with yellow spheres, and Se with green spheres.

The structure of the studied TaSSe as well as of its parent compounds, TaS_2_ and TaSe_2_, is shown in Figure 1, presenting side and top views of all the structures, with an in-plane unit cell marked with a solid rhombus (with the side length equal to the lattice constant *a*). The values of structural parameters predicted by the present DFT calculations are summarized in Table 1, containing the lattice constants, the monolayer thickness *d* (defined as the distance between the upper and the lower plane containing chalcogen atoms), the relevant bond lengths between Ta atom and chalcogen atoms $d_{\mathrm{Ta}-S}$ and $d_{\mathrm{Ta}-\mathrm{Se}}$, as well as the bond angles. The meaning of all the parameters is explained in a detailed way in Figure 2 (see [93]).

In general, a good consistency between the calculated and the measured lattice parameter *a* values can be found, with the trend that the lattice constant for TaSe_2_ is larger than for TaS_2_ (as the atomic radius is larger for Se than for S); for the Janus structure, the predicted *a* value is actually exactly an average of the lattice constants of the parent compounds. Also, the layer thickness takes a larger value for Se-containing TMD than for S-based TMD. In Table 1, we offer some comparison with the experimental results. It must be emphasized that the experimental values after Ref. [57] concern XRD measurements performed on the bulk samples. Moreover, the TaSSe data in Ref. [57] do not correspond to the Janus structure, but to TaS_2−x_Se_x_ random alloy with x=1. Therefore, such quantities as the bond lengths and the bond angles would be regarded as the averages of relevant parameters for Ta-S and Ta-Se bonds in the TaSSe Janus structure. On the other hand, the values reported in Ref. [94] relate to the monolayers (but they result from the STM topography measurements, thus having significantly larger uncertainties than the XRD-based ones).

**Figure 3:**
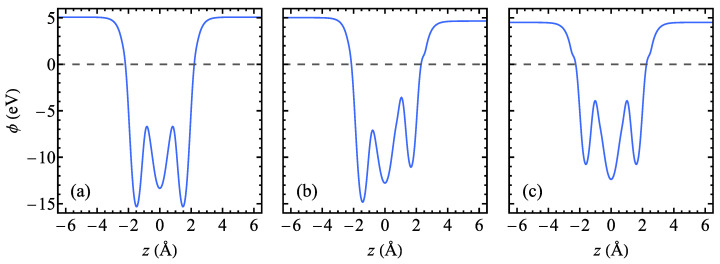
The planar-averaged electrostatic potential as a function of the co-ordinate perpendicular to the layer for a normal phase of 1T polymorph of (**a**) TaS_2_, (**b**) TaSSe, and (**c**) TaSe_2_ monolayer as predicted by the DFT calculations. The Fermi level is marked with a dashed line.

An interesting quantity for two-dimensional systems is the planar-averaged electrostatic potential as a function of the co-ordinate normal to the surface. It gives information about the electric field distribution inside the structure. The planar-averaged electrostatic potential ϕ is plotted as a function of the co-ordinate *z* perpendicular to the heterostructure plane in Figure 3 for the studied Janus TaSSe and for the reference structures, TaS_2_ and TaSe_2_. The value of z=0 corresponds to the position of the plane of Ta atoms. The asymmetric shape of the function ϕ(z) with two deep minima of unequal depth, corresponding to the planes of S and Se atoms, marks the presence of an in-built electric field in the Janus heterostructure (Figure 3b), whereas for TaS_2_ (Figure 3a) and TaSe_2_ (Figure 3c), the analogous functions are symmetric (even) and no such in-built field is present. The difference in electrostatic potential at the position of S and Se plane can be estimated as 3.77 eV, being considerably larger than those predicted for PtSSe or other Pt-based Janus monolayers [95], WSSe [96], or MnSSe [97]. The presence of an intrinsic electric field is a distinct feature of the Janus structures, leading to the interesting physical properties. As an example, the mirror symmetry breaking and the presence of an intrinsic electric field lead to inducing of the Rashba spin–orbit coupling [8,98].

The value of the potential ϕ at a large distance from the surface relative to the Fermi level is equal to the work function of the material $\phi_{WF}$. The values of predicted work functions are collected in Table 2. It is notable that for Janus TaSSe, the values of work function at both sides of the layer differ by 0.368 eV, being a manifestation of an in-built electric field. This value is slightly smaller than the one predicted for MoSSe and WSSe [18,99] or PtSSe [95]. On the other hand, when the values of $\phi_{WF}$ for TaSSe at each side are compared with $\phi_{WF}$ for TaS_2_ and TaSe_2_, it follows that the value at the S side is slightly lower with respect to the TaS_2_ case, whereas the value at the Se side indicates an opposite tendency when compared with the result for TaSe_2_. The work function values for TaS_2_ are in accordance with the experimental results for bulk 1T-TaS_2_ [100]. In addition, it should be noted that a recent calculation of work function for monolayer 1T-TaSe_2_ gave values consistent with our results [101].

The in-built electric field in the Janus structure stemming from the difference in electronegativity between the chalcogen atoms at both sides is connected with the emergence of an electric dipole moment μ, which can be calculated within the DFT formalism. In TaSSe, the dipole points from the S layer to the Se layer and its value is predicted to be equal to 0.0205 eV·Å (or 0.0983 Debye), whereas no dipole moment emerges for the parent compounds, TaS_2_ and TaSe_2_. The value obtained for TaSSe is somehow smaller than the one calculated by the DFT, for example, for MoSSe [18,99,102], WSSe [18,99], PtSSe [95,103], or RhSSe [104].

It is particularly interesting to investigate the effect of an external, perpendicular electric field on the selected quantities characterizing a Janus TaSSe monolayer. Similar computational studies in the literature involved such Janus monolayers as MnSSe [97], SnSSe [105], MoSSe [102], PtSSe [106], or HfSSe [107].

**Figure 4:**
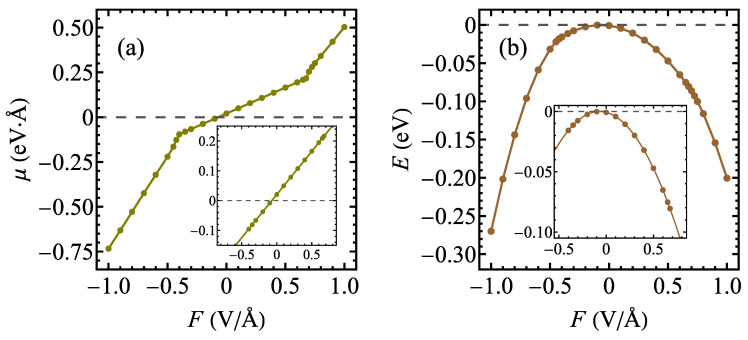
The dependence of the electric dipole moment (**a**) and the total energy (**b**) on the external electric field for a normal phase of 1T polymorph of TaSSe monolayer as predicted by the DFT calculations. The insets focus on the field range between −0.4 and 0.675 V/Å and show the analytic dependencies fitted to the DFT data according to Equation (1) for (**a**) and Equation (2) for (**b**) with solid lines.

The dependence of the electric dipole moment on the external electric field *F* is shown in Figure 4a. Note that the positive field in our convention points from the S plane to the Se plane. The observed dependence is piecewise linear. The inset shows the magnified region of the linear dependence of μ on *F* in the range of fields between −0.40 and 0.675 V/Å. In this particularly interesting range, a linear function,

(1) $$\mu =\alpha \left(F-F_{0}\right),$$

can be fitted to the DFT data, resulting in estimation of the polarizability α equal to 0.290 e·${Å}^{2}$/V (or 4.17 ${Å}^{3}$ if expressed as the polarizability volume). The electric dipole moment vanishes when the external field $F_{0}$ of −0.0705 V/Å is applied (i.e., the in-build electric field is compensated by the external field equal to $F_{0}$).

It is instructive to additionally analyze the total energy of the structure obtained from the DFT calculations as a function of the external electric field, as shown in Figure 4b. The energy scale is adjusted such that the maximum energy equals 0. The position of the maximum is exactly consistent with the vanishing of the dipole moment. A similar effect can be noticed, for example, for MoSSe [102]. The energy of the induced electric dipole in an external electric field can be written as E=−μF/2, finally yielding

(2) $$E=-\frac{1}{2}\alpha F\left(F-F_{0}\right)$$

if the induced dipole moment μ is expressed by Equation (1). This parabolic approximation is valid in the range of electric fields between −0.4 and 0.675 V/Å, and the relevant parabolic function with α and $F_{0}$ determined from fitting Equation (1) to the DFT data for a dipole moment is shown in the inset in Figure 4b, showing excellent agreement with the DFT data.

The charge redistribution in the structure can be analyzed based on the Bader charge [108], like in other Janus monolayers [97,109,110]. The values of Bader charge transfer on each constituent atom for TaSSe and its parent compounds are collected in Table 3 (the values of charge transfer are relative to the thirteen valence electrons of Ta and the six valence electrons for S and Se). The results indicate that the electrons are transferred from the Ta atom to chalcogen atoms. As the electronegativity of S is slightly larger than that of Se, the Bader charge of the S atom exceeds somewhat the one attributed to the Se atom. Moreover, the electron gain of a given chalcogen atom (S or Se) is similar in a Janus structure and in a non-Janus typical dichalcogenide system (however, S in TaSSe gains slightly more electrons than in TaS_2_, while the opposite is true for Se). The unequal value of the charge transfer for S and Se atoms results in the emergence of the in-built electric field in a Janus structure.

**Figure 5:**
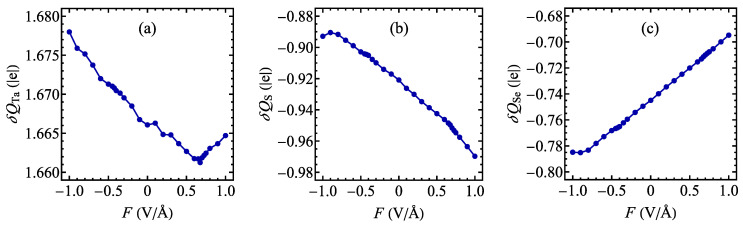
The Bader charge transfer for Ta (**a**), S (**b**), and Se (**c**) atoms as a function of the external electric field for a normal phase of 1T polymorph of TaSSe monolayer as predicted by the DFT calculations.

It is also interesting to analyze the Bader charge transfer dependence on the external electric field (see [97]), which is plotted in Figure 5. The results show an approximately linearly decreasing Bader charge as a function of the field for the range of fields below 0.675 V/Å for Ta atoms (Figure 5a), whereas the trend is reversed for the stronger fields. For the chalcogen atoms, S tends to lower its Bader charge if the field is increased (with the exception of strong negative fields; see Figure 5b), while an exactly opposite tendency is visible for Se atoms (Figure 5c). Therefore, the field pointing from the S plane to the Se plane tends to push the electrons from Se to S atoms. It should be observed that the magnitude of the field-induced variation in Bader charge for the Ta atom is significantly lower than for the chalcogen atoms, so that the field mainly causes the charge transfer between both chalcogen layers.

**Figure 6:**
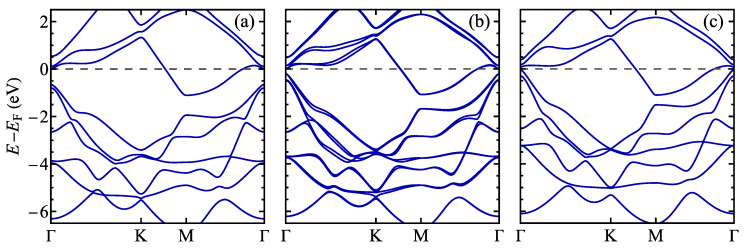
The electronic structure of a normal phase of 1T polymorph of (**a**) TaS_2_, (**b**) TaSSe, and (**c**) TaSe_2_ monolayer as predicted by the DFT calculations. The Fermi level is set to 0 and marked with a dashed line.

**Figure A1:**
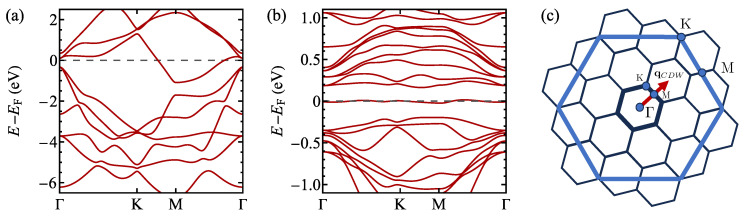
The electronic structure of a normal phase (**a**) and of a distorted phase (**b**) of 1T polymorph of TaSSe monolayer as predicted by the DFT calculations without taking into account spin-orbit coupling. The Fermi level is set to 0 and marked with a dashed line. The first Brillouin zones are shown in (**c**): the large hexagon marks a zone for the normal phase, whereas the small hexagons represent the zones for the distorted phase. The special points are marked with capital letters. The CDW wavevector $q_{CDW}$ is shown with an arrow.

The electronic structure of TaSSe and its parent compounds in the absence of an external electric field can be viewed in Figure 6 (where the Fermi level is set to zero and marked with a dashed line), showing the calculation along the Γ–K–M–Γ path in the first Brillouin zone (see Figure A1c). In all the cases, the monolayer has metallic properties, with a dispersive band crossing the Fermi level in between the K and the M high-symmetry point of the first Brillouin zone. This contrasts with such monolayers as MoSSe [18,99,102], WSSe [18,99], or PtSSe [95,103], which exhibit significant energy gaps in the band structures. A notable feature of TaSSe is lifting the band degeneracy resulting in band splitting due to the mirror symmetry breaking, which results in the presence of spin–orbit coupling. A result of the corresponding calculation without taking into account the spin–orbit coupling can be viewed in Figure A1a, where no band splitting occurs. The TaSSe band structure generally interpolates between TaS_2_ and TaSe_2_ cases. It is particularly visible for the Γ point and the position of valence-like bands below the Fermi level; the bands are separated by a gap from the conduction-like band touching the Fermi level for TaS_2_, whereas they almost merge for TaSe_2_; for the Janus structure, we deal with an intermediate gap value.

**Figure 7:**
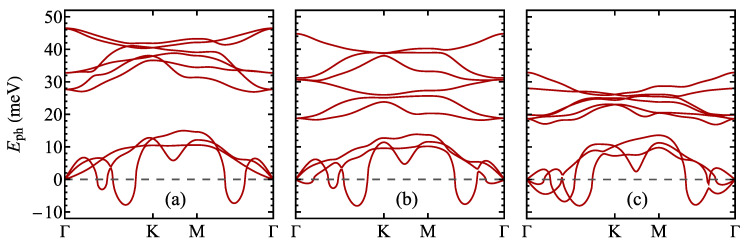
The phonon energies for a normal phase of 1T polytype of (**a**) TaS_2_, (**b**) TaSeS, and (**c**) TaSe_2_ monolayer as predicted by the DFT calculations. The negative values (below the dashed line) correspond to the imaginary modes.

The characterization of the physical properties of monolayer TaSSe in normal (undistorted) phase can be supplemented with the calculation of phonon energies, the result of which is shown in Figure 7 (for the case of TaSSe as well as its parent compounds). It should be emphasized that in the data presentation, the negative values correspond to the imaginary frequencies of phonon modes, giving rise to a structural instability. A striking feature of all the results is the presence of evident imaginary frequency modes along the Γ–K and Γ–M path. It should be emphasized that the most pronounced imaginary frequency mode appears in our calculations for a similar wavevector in all three cases—TaS_2_, TaSSe, and TaSe_2_. Such a situation is responsible for the instability of the systems towards a structural distortion causing PLD and entailing the emergence of CDW. Moreover, the close correspondence between the wavevectors at which the most pronounced imaginary modes occur and the characteristic wavevectors of CDW can be found, as emphasized, for example, by [111] for the bulk case or by [112] for the monolayer case of TaS_2_. The characteristic wavevector of CDW phase for the $\sqrt{13}\times \sqrt{13}$ reconstruction is marked in Figure A1c, showing the relevant first Brillouin zones. The vector is rotated anticlockwise by $13.9^{\circ }$ from the Γ–M direction and its magnitude is equal approximately to 0.555 of the distance from Γ to M point. Let us mention here the calculations of phonon dispersion relations for monolayer TaS_2_ [30,33,113] and TaSe_2_ [33,114] existing in the literature.

**Figure 8:**
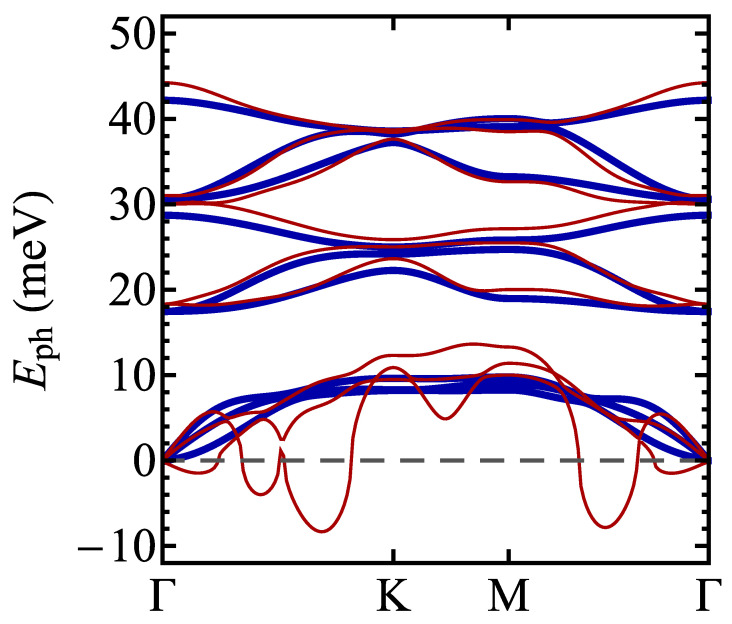
The phonon energies for a normal phase of 1T polymorph of TaSSe monolayer as predicted by the DFT calculations with Fermi–Dirac smearing for the smearing temperature of 6000 K (thick lines) and 100 K (thin lines). The negative values correspond to the imaginary modes.

In order to simulate the influence of the increasing temperature on the phonon energies, we have performed the relevant DFT calculations using Fermi–Dirac smearing (see, for example, Ref. [114] or [115]). Figure 8 shows the phononic dispersion relation obtained for TaSSe for the smearing temperature of 6000 K (thick lines) and for 100 K (thin lines). Let us emphasize that the electronic temperature used as a parameter for smearing relates only to the electronic subsystem, not to the ionic one, and is not equivalent to the physical equilibrium temperature of the studied system. Therefore, it is used to qualitatively illustrate the mechanism of thermally induced changes in the phononic frequencies. It is visible that the imaginary modes related to the acoustic phonons at low temperatures vanish when the temperature is elevated, so that the undistorted structure becomes stabilized by the increasing temperature. This mechanism is similar to the one observed in TiSe_2_ in Ref. [114].

### 3.2. Distorted Phase

**Figure 9:**
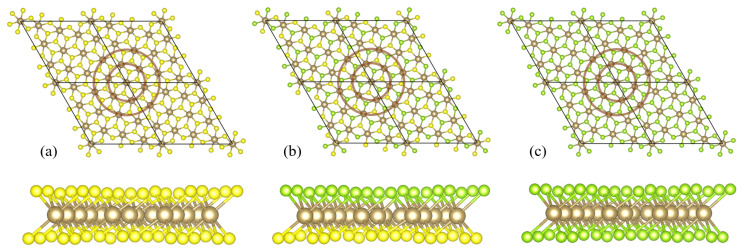
The top views of 4 supercells and the side views of single supercells of the crystalline structure of a distorted phase of 1T polymorph of (**a**) TaS_2_, (**b**) TaSSe, and (**c**) TaSe_2_ monolayer. The unit cells are marked with a thick solid line in the top view. The Ta atoms are marked with brown spheres, S with yellow spheres, and Se with green spheres. The brown circles mark an inner ring and an outer ring of Ta atoms surrounding the central (unshifted) atom of a star-of-David deformation.

After a thorough analysis of the normal phase properties, we present a discussion focused on the results obtained for a $\sqrt{13}\times \sqrt{13}$ supercell. The supercell geometry involves its rotation with respect to the 1 × 1 cell by an angle of $13.9^{\circ }$. For such a system, the atomic position relaxation leads to a distorted phase with PLD, giving rise to a commensurate CDW for all the three studied compounds. A top and side view of structures obtained for the supercell calculations is shown in Figure 9. In the top view, four supercells are shown (each marked with a solid rhombus). A distinct feature of PLD in the discussed class of TMD is the star-of-David shape of the cluster formed by Ta atoms (which experience the most pronounced in-plane shifts). A supercell contains 13 Ta atoms; the central Ta atom of the star (situated at the edge of the supercell in our calculations) remains unshifted. An inner ring and an outer ring of Ta atoms (containing six atoms each and marked with solid circles in Figure 9) can be singled out. The radii of the rings are reduced in PLD phase with respect to the values in normal phase, and this behavior is common to TaSSe and its parent compounds. The effect can be quantitatively tracked using the data collected in Table 4, where the radii of the inner ring ($r^{\left(1\right)}$) and outer ring ($r^{\left(2\right)}$) are given for both phases, together with radii differences $\Delta r^{\left(1\right)}$ and $\Delta r^{\left(2\right)}$ between the phases. It is visible that the magnitudes of radius reduction in TaSSe are close to the values predicted for TaS_2_, whereas the values found for TaSe_2_ are lower (all the relative magnitudes being at the level of a few percent).

The values of the difference in total energy between the normal phase (without PLD) and the CDW phase (with PLD), calculated in a $\sqrt{13}\times \sqrt{13}$ supercell and normalized per formula unit, are collected in Table 5. The results are expressed both in energy units and in temperature units (the latter being a sort of rough estimate of the transition temperature). It is notable that in all the cases, the CDW phase has significantly lower energy than the normal phase; the energetic stability of the CDW phase in TaSSe might be comparable to the case of TaS_2_. It is worth mentioning here that the transition temperature for monolayer TaSe_2_ was experimentally determined as 530 K [46] (while it amounts to 180 K for bulk TaS_2_ according to the same source).

**Figure 10:**
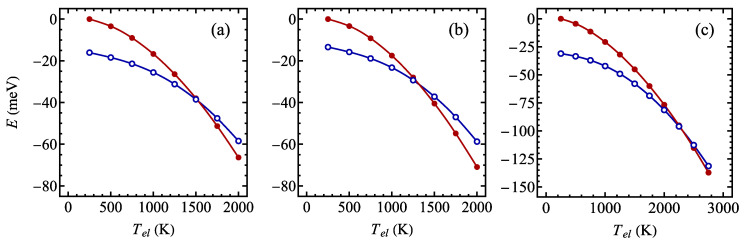
The total energy per supercell of a normal phase (filled circles) and a CDW phase (empty circles) of 1T polymorph of (**a**) TaS_2_, (**b**) TaSSe, and (**c**) TaSe_2_ monolayer as a function of the smearing temperature as predicted by the scalar relativistic DFT calculations with Fermi–Dirac smearing.

In order to illustrate the suggested phase transition between a CDW and a normal phase when the temperature is increased, we additionally studied the dependence of the total energy per formula unit on the smearing temperature used in the DFT calculations (using Fermi–Dirac smearing for the scalar relativistic calculations). A similar approach was adopted in Ref. [115] for the case of TiSe_2_. The results for TaSSe and its parent compounds are shown in Figure 10. In all the cases, for low smearing temperatures, the phase with CDW has significantly lower energy than the undistorted phase. When the smearing temperature is increased, the energy difference between the phases diminishes and, above some critical smearing temperature, the lowest energy phase is the undistorted phase. This sort of behavior indicates that the low-temperature stable phase should be a CDW phase.

In the side view of the structures in Figure 9, some buckling of the Ta plane can also be observed, followed by a more distinct buckling of the upper and lower planes of chalcogen atoms.

**Figure 11:**
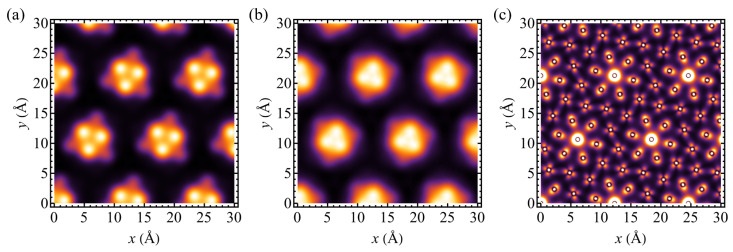
The local density of states (in arbitrary units) for CDW phase of a TaSSe monolayer, calculated at the Fermi level with the energy window width of 0.1 eV, for the plane located 3 Å below the outermost atom from S layer (**a**), for the plane located 3.5 Å over the outermost atom from Se layer (**b**), and for the plane corresponding to the position of Ta atoms (**c**).

The manifestation of buckling of the chalcogen planes is a non-uniform pattern of the local density of states (LDOS) in CDW phase. A calculated LDOS map for TaSSe is shown in Figure 11 for the energy window centered at the Fermi level and having a width of 0.1 eV; the map covers a square 30 × 30 ${Å}^{2}$. Figure 11a presents a LDOS map for the plane located 3 Å below the outermost atom from the S layer, whereas Figure 11b presents a map for the plane located 3.5 Å over the outermost atom from Se layer. In both plots, the characteristic triangular patterns contributed by the chalcogen atom states are visible, as in the case of the parent compounds TaS_2_ and TaSe_2_ and other $\sqrt{13}\times \sqrt{13}$ supercell-forming TMDs [34,36,46,48,94,116,117,118,119]. Such a modelling can be qualitatively compared to the STM image and verified experimentally [34]. It is visible that our results indicate the presence the of pattern characteristic of a CDW phase with star-of-David clusters.

**Figure 12:**
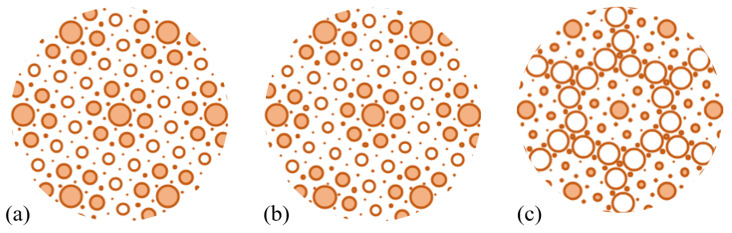
The visualization of the difference in Bader charge between the distorted and the normal phase of a 1T polymorph of (**a**) TaS_2_, (**b**) TaSSe, and (**c**) TaSe_2_ monolayer. The filled circles correspond to the gain of electrons, while empty ones denote the loss of electrons by atoms; the absolute value of charge difference is proportional to the circle radius; note the different scale for each panel.

The emergence of PLD is connected with the formation of CDW. The charge redistribution can be tracked first on the basis of the Bader charge transfer for all three studied compounds. Table 6 summarizes the Bader charge transfer values (charge relative to the number of valence electrons of a given atom) for the central Ta atom of a star-of-David deformation ($\delta Q_{\mathrm{Ta}}^{\left(0\right)}$), for Ta atoms from the inner ring ($\delta Q_{\mathrm{Ta}}^{\left(1\right)}$) and from the outer ring ($\delta Q_{\mathrm{Ta}}^{\left(2\right)}$). Moreover, the average values for Ta, S, and Se atoms are given ($\langle \delta Q_{\mathrm{Ta}}\rangle$, $\langle \delta Q_{S}\rangle$, and $\langle \delta Q_{\mathrm{Se}}\rangle$, respectively). The average values are considerably close to the values for the normal phase, collected in Table 3. It can be concluded that the charge redistribution during the formation of a CDW phase takes place mainly among the Ta atoms, whereas the contribution of the chalcogen atoms is less important. Namely, the central Ta atom gains the electrons and so do the Ta atoms from the inner ring, whereas the Ta atoms from the outer ring become depleted of electrons. The process is visualized in Figure 12, which shows the differences in Bader charge values between the CDW phase and the normal phase. In the figure, the plots are centered at the unshifted Ta atom of a star-of-David distortion (located at the edge of the supercell), and the radii of discs are proportional to the Bader charge difference (with different scale for each compound shown in panels Figure 12a–c). In the case of TaSSe (Figure 12b) and TaS_2_ (Figure 12a), the central Ta atom gains most of the electrons (also, the inner ring of Ta atoms is enriched with electrons). For TaSe_2_ (Figure 12c), the charge gain by the central Ta atom is even more pronounced when compared to the inner ring of Ta atoms, whereas the outer ring loses a more significant number of the electrons (which are also redistributed to Se atoms in this case). The calculations support the picture of the central, unshifted Ta atom gaining the charge [24,37,120,121] under the formation of a CDW phase. The picture of charge gain by the central Ta atom is additionally supported by the calculation of LDOS at the Fermi level for the plane containing Ta atoms, as shown in Figure 11c. There, the global LDOS maxima correspond to the positions of central Ta atoms, proving the tendency towards charge localization at these positions.

**Figure 13:**
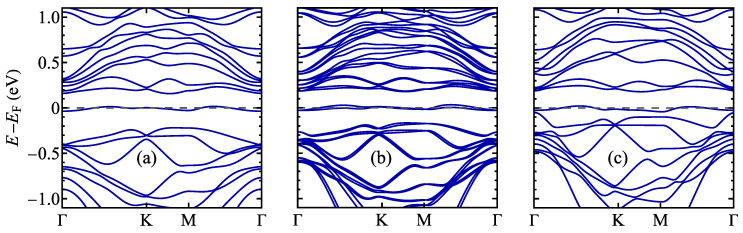
The electronic structure of the distorted phase of a 1T polymorph of (**a**) TaS_2_, (**b**) TaSSe, and (**c**) TaSe_2_ monolayer. The Fermi level is set to 0 and marked with a dashed line.

The band structure calculated for a $\sqrt{13}\times \sqrt{13}$ supercell for TaSSe and its parent compounds is shown in Figure 13, along the Γ–K–M–Γ path in the supercell’s first Brillouin zone (note the difference between the supercell and the 1 × 1 cell case, as illustrated in Figure A1c). A notable feature of all three plots is the presence of a weakly dispersive, almost flat band located at the Fermi level, which was not present for the undistorted structures (Figure 6) and has a significant meaning for the CDW physics [28,122,123]. This band is contributed mainly by *d* orbitals of Ta atoms, mostly by the central, unshifted Ta atom of a star-of-David cluster [28,123]. The flat band is separated from the conduction-like bands with a significant gap. For TaS_2_ and Janus TaSSe, the valence-like bands also lie considerably lower in energy than the flat band, whereas for TaSe_2_, the mentioned bands almost merge at the Γ point. Like in the undistorted phase case, the band for TaSSe is additionally split due to the mirror symmetry breaking causing a spin–orbit coupling. As a reference, the band structure calculated without accounting for spin–orbit coupling is shown in Figure A1b; no band splitting is visible in these results.

**Figure 14:**
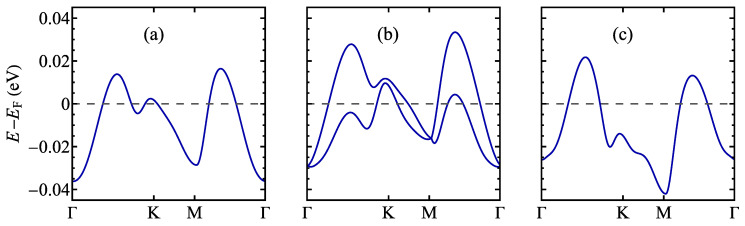
The electronic structure of the distorted phase of a 1T polymorph of (**a**) TaS_2_, (**b**) TaSSe, and (**c**) TaSe_2_ monolayer in the vicinity of the Fermi level (which is set to 0 and marked with a dashed line).

The band structure close to the Fermi level is plotted separately in Figure 14. For TaS_2_ and TaSe_2_, there is a band minimum at the Γ point, whereas for Janus structure TaSSe, this minimum is shifted away from the Γ point, supporting the picture of the importance of Rashba spin–orbit coupling for the band structure.

**Figure 15:**
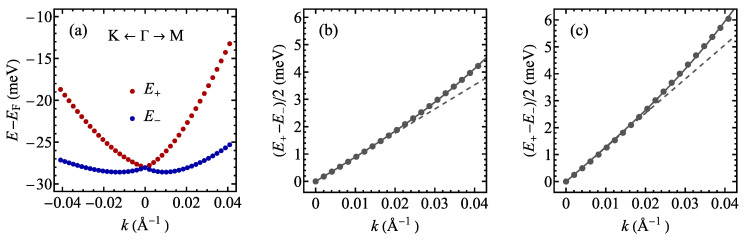
The detailed behavior of the weakly dispersive split band for the distorted phase of a TaSSe monolayer near the Fermi level in the vicinity of the Γ point along the path towards K point (negative *k*) and towards M point (positive *k*) (**a**). The difference in energy between $E_{+}$ and $E_{-}$ divided by two as a function of the wavevector in the vicinity of the Γ point, along the path to the K point (**b**) and to the M point (**c**). The points mark the results of the DFT calculations, whereas the solid lines denote the fitted function from Equation (2) and the dashed lines correspond to the same model but with $\alpha_{3}=0$.

Focusing on the weakly dispersive bands at the Fermi level, we have performed the calculations of the dispersion relations close to the Γ point in the directions Γ–K and Γ–M (as shown in Figure 15a). A clear shift of the band minima from the Γ point (k=0) to the non-zero wavevector $k_{R}$ can be observed in the data (with an anisotropic behavior in the wavevector space), suggesting the presence of Rashba spin–orbit coupling. The minima correspond to the energy $E_{R}=E\left(k=0\right)-E\left(k_{R}\right)$. This sort of behavior is expected in Janus TMD monolayers due to an in-built electric field perpendicular to the layer, owing to the different electronegativity of chalcogen atoms in the top and bottom layers and to the mirror symmetry breaking. Moreover, a non-parabolic dispersion is clearly visible for both bands, suggesting the presence of the cubic Rashba spin–orbit coupling contribution in addition to the usual linear term. In order to quantify the Rashba spin–orbit coupling, we have fitted our DFT data for the difference between upper and lower band energies (see Figure 15a) with the following equation [124,125,126,127,128,129]:

(3) $$E_{+}\left(k\right)-E_{-}\left(k\right)=2\left(\alpha_{1}k+\alpha_{3}k^{3}\right),$$

with Rashba coefficients $\alpha_{1}$ and $\alpha_{3}$. The results are collected in Table 7 for the directions Γ–K and Γ–M. The fit quality can be assessed on the basis of Figure 15b,c, where the solid lines show the fitted model with $\alpha_{1}$ and $\alpha_{3}$, the dashed lines correspond to the model with $\alpha_{3}=0$, and the points mark our DFT data. The data for $\alpha_{1}$ could be compared with the calculations presented in Ref. [130] for the other Janus monolayer TMDs. It can be observed that $\alpha_{1}$ values are close to the ones predicted for MoSSe and WSSe, with somehow more pronounced anisotropy in the wavevector space for our case of TaSSe.

**Figure 16:**
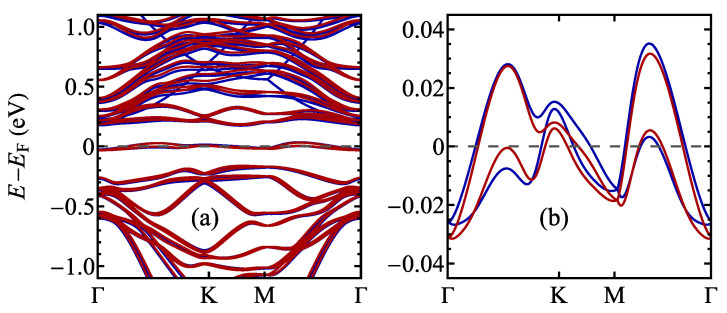
The electronic structure of the distorted phase of a 1T polymorph of TaSSe monolayer for the external field of 0.4 V/Å (red lines) and −0.4 V/Å (blue lines), for the larger energy range (**a**) and for the energy range focusing on the weakly dispersive band close to the Fermi level (**b**). The Fermi level is set to 0 and marked with a dashed line.

In order to illustrate the influence of the electric field on the CDW state of TaSSe monolayer, in Figure 16, we show its band structure calculated for the field values of ±0.4 V/Å. The general band structure, visualized in Figure 16a, shows that the influence of the electric field on the bands below the Fermi level is not significant in this energy scale. On the contrary, above the Fermi level, some additional bands appear for the negative field; moreover, the energy differences between both cases are more pronounced. In order to focus on the weakly dispersive band close to the Fermi level, we present Figure 16b, which confirms that for both studied electric field values, the flat band behaves in a similar manner as in the absence of the field (see Figure 14b).

It can be mentioned here that our calculations involve only a monolayer Janus system. The physics of monolayer and bulk TMDs (such as TaS_2_) differs, for example, at the level of the band structure (see, for example, Ref. [31]). For instance, in the CDW phase of bulk TaS_2_, a band exhibiting the dispersion along an out-of-plane direction is present [31,118], contrary to the monolayer case. Moreover, in multilayer (or bulk) TMDs such as TaS_2_, various stackings of the individual layers are possible. This can take place at two levels. The first one is connected with the mutual position of TMD monolayers in (undistorted) multilayer (see, for example, the various geometries investigated in Ref. [131] or Ref. [132] for W- and Mo-based Janus TMDs). The second one is related to the relative phase of CDW in monolayers composing a multilayer system [23,133,134,135,136,137,138,139]. These possibilities give rise to yet another factor shaping the already complex physical picture in the systems and enriching the phase diagram due to interlayer interactions. The stacking-related degree of freedom is even proved to provide the possibility to engineer the CDWs in TaS_2_ by stackingtronics [113]. As a consequence, a similar situation could also be envisaged in Janus TaSSe systems composed of more than one layer. Furthermore, the physics of Janus multilayers would be enriched in comparison to the parent compounds due to the presence of an in-built electric dipole and its thickness dependence. Nevertheless, it must be emphasized that for TaS_2_ and TaSe_2_, both bulk and monolayer systems exhibit the experimentally confirmed presence of CDWs with the same $\sqrt{13}\times \sqrt{13}$ reconstruction. Some examples of the effect of transition from monolayer to multilayer structure for Janus TMDs are presented in Refs. [131,132], where such parameters as the Rashba spin–orbit coupling coefficient or the in-built dipole are influenced.

## 4. Conclusions

We studied the properties of a normal and a CDW phase of 1T-TaSSe Janus monolayer using the DFT formalism, comparing the results with the outcome of calculations carried out for TaS_2_ and TaSe_2_. Two parent compounds, TaS_2_ and TaSe_2_, are predicted theoretically and confirmed experimentally to exhibit a $\sqrt{13}\times \sqrt{13}$ CDW, both in the bulk and in the monolayer form.

For the normal state, we find that the structural parameters of TaSSe interpolate between the relevant properties of the parent compounds. The broken mirror symmetry for the Janus structure has numerous physical implications, one of them being the emergence of an in-built electric field in the structure and appearance of a non-zero electric dipole moment. We investigated the behavior of this moment in the external electric field normal to the monolayer plane, finding a piecewise linear behavior and estimating the polarizability. In addition, we studied the dependence of the total energy on the electric field, finding consistency with the behavior of an induced electric dipole. The charge transfer within the Janus monolayer and its dependence on the electric field was quantified using the Bader charge analysis. The calculations of the band structure revealed the metallic nature of the studied monolayers with a dispersive band crossing the Fermi level. For Janus TaSSe, a band splitting resulting from mirror symmetry breaking is found. The analysis of phonon dispersion relations predicted the presence of the robust modes of imaginary energy, responsible for a structural instability of the normal phase with respect to the formation of PLD.

The phase with CDW was studied for a $\sqrt{13}\times \sqrt{13}$ reconstruction. The energetic stability of the phase with CDW was stated for all the studied structures (where the energy difference between the CDW and the normal state for TaSSe is comparable with the value for TaS_2_) and the geometry of the structure deformation was described. The formation of CDW was confirmed by the analysis of Bader charge transfer as well as by the calculation of LDOS maps, predicting behavior close to that exhibited by the parent compounds for each side of the Janus structure. The band structure of the CDW state was demonstrated to develop a weakly dispersive, flat band at the Fermi level. For TaSSe, this band is split owing to the Rashba spin–orbit coupling emerging due to an in-built electric field and a mirror symmetry breaking [98]. The Rashba spin–orbit coupling was then quantified with the linear and the cubic terms with the wavevector space anisotropy in the vicinity of the Γ point.

Our results predict that TaSSe is a potentially promising platform for studying CDW in Janus structures, where a subtle interplay of the complex physics of CDWs and the effects caused by the symmetry breaking can be expected. This can be exemplified by the presence of a flat band at the Fermi level with a noticeable splitting due to the spin–orbit coupling. Moreover, the properties of Janus TaSSe show a pronounced sensitivity to the external electric field, providing an additional tuning knob to control the system properties in a reversible way. In this context, the recent achievements in obtaining a noticeably intensive electric field using a dual ionic gating can be emphasized [140,141], furnishing a tool to study the field-induced behavior. Further developments may include exploring the effect of point defects on the studied structure (see [142] for Janus structures), as such defects occur in TMDs and shape their properties [143,144]; they were recently shown to influence the CDWs in TaS_2_ [34,145]. Other directions involve further studies on the control of CDWs with the electric field [146] or the strain [29].

## Figures and Tables

**Table 1:** The structural parameters of monolayers of a 1T polymorph of TaS_2_, TaSSe, and TaSe_2_ in normal phase as predicted by the DFT calculations together with some relevant experimental values taken from Refs. [57,94].

Parameter	TaS_2_	TaSSe	TaSe_2_
*a* (Å)	3.345 (exp.: 3.367 [57]; 3.38 [94])	3.408 (exp.: 3.417 [57])	3.469 (exp.: 3.475 [57]; 3.44 [94])
*d* (Å)	3.029	3.139	3.257
$d_{\mathrm{Ta}-S}$ (Å)	2.454 (exp.: 2.441 [57])	2.453 (exp.: 2.501 [57])	–
$d_{\mathrm{Ta}-\mathrm{Se}}$ (Å)	–	2.584 (exp.: 2.501 [57])	2.581 (exp.: 2.547 [57])
$\alpha_{S-S}$${(}^{\circ }$)	85.93 (exp.: 87.23 [57])	88.01 (exp.: 86.20 [57])	–
$\alpha_{\mathrm{Se}-\mathrm{Se}}$${(}^{\circ }$)	–	82.54 (exp.: 86.20 [57])	84.43 (exp.: 86.01 [57])
β${(}^{\circ }$)	94.07	94.67	95.57

**Table 2:** The work function of monolayers of a 1T polymorph of TaS_2_, TaSSe, and TaSe_2_ in normal phase, as predicted by the DFT calculations together with the relevant experimental value taken from Ref. [100].

Parameter	TaS_2_	TaSSe	TaSe_2_
$\varphi_{WF}$ (eV), S side	5.078 (exp. 5.2 [100])	5.023	–
$\varphi_{WF}$ (eV), Se side	–	4.655	4.509

**Table 3:** The Bader charge transfer for the atoms in monolayers of a 1T polymorph of TaS_2_, TaSSe, and TaSe_2_ in normal phase, as predicted by the DFT calculations.

Parameter	TaS_2_	TaSSe	TaSe_2_
$\delta Q_{\;\mathrm{Ta}}$ (|e|)	1.8073	1.6661	1.5278
$\delta Q_{\;S}$ (|e|)	−0.9037	−0.9209	
$\delta Q_{\;\mathrm{Se}}$ (|e|)	–	−0.7450	−0.7638

**Table 4:** The parameters characterizing the radii of rings of Ta atoms centered at the central atom in a star-of-David cluster for a normal phase and a CDW phase of 1T polymorph of TaS_2_, TaSSe, and TaSe_2_ monolayer as predicted by the DFT calculations.

Parameter	TaS_2_	TaSSe	TaSe_2_
$r^{\left(1\right)}$, normal phase (Å)	3.345	3.408	3.469
$r^{\left(1\right)}$, CDW phase (Å)	3.178	3.238	3.308
$\Delta r^{\left(1\right)}$ (Å)	−0.167	−0.171	−0.161
$r^{\left(2\right)}$, normal phase (Å)	5.794	5.903	6.008
$r^{\left(2\right)}$, CDW phase (Å)	5.606	5.718	5.844
$\Delta r^{\left(2\right)}$ (Å)	−0.188	−0.185	−0.165

**Table 5:** The energy difference per formula unit between normal phase and CDW phase for a $\sqrt{13}\times \sqrt{13}$ supercell as predicted by the DFT calculations.

Parameter	TaS_2_	TaSSe	TaSe_2_
ΔE (meV)	17.7	15.6	58.7
$\Delta E/k_{B}$ (K)	205	181	681

**Table 6:** The Bader charge transfer for Ta, S, and Se atoms for a CDW phase of 1T polymorph of TaS_2_, TaSSe, and TaSe_2_ monolayer as predicted by the DFT calculations.

Parameter	TaS_2_	TaSSe	TaSe_2_
$\delta Q_{\mathrm{Ta}}^{\left(0\right)}$ (|e|)	1.634	1.510	1.395
$\delta Q_{\mathrm{Ta}}^{\left(1\right)}$ (|e|)	1.703	1.577	1.460
$\delta Q_{\mathrm{Ta}}^{\left(2\right)}$ (|e|)	1.912	1.771	1.629
$\langle \delta Q_{\mathrm{Ta}}\rangle$ (|e|)	1.794	1.661	1.533
$\langle \delta Q_{S}\rangle$ (|e|)	−0.897	−0.918	
$\langle \delta Q_{\mathrm{Se}}\rangle$ (|e|)		−0.744	−0.766

**Table 7:** The parameters characterizing Rashba spin–orbit coupling for TaSSe weakly dispersive bands at the Fermi level in the distorted phase as predicted by the DFT calculations.

Direction	$k_{R}$ ($10^{-3}$${Å}^{-1}$)	$E_{R}$ (meV)	$\alpha_{1}$ (meV·Å)	$\alpha_{3}$ (eV·${Å}^{3}$)
Γ–K	13.0	0.536	88.2	9.2
Γ–M	9.40	0.535	127	13.6

## Data Availability

The original contributions presented in the study are included in the article, further inquiries can be directed to the corresponding author.

## Abbreviations

The following abbreviations are used in this manuscript:

CDW	Charge density wave
DFT	Density functional theory
LDOS	Local density of states
PLD	Periodic lattice distortion
TMD	Transition metal dichalcogenide

## Appendix A. Electronic Structure Calculations without Spin-Orbit Coupling

An important factor shaping the electronic structure of Janus TMDs is the spin-orbit coupling. Our main DFT calculations included this factor and were based on a non-collinear, fully relativistic approach. As a reference, we show in Figure A1a,b the band structure of a normal and a distorted phase of 1T-TaSSe calculated in the collinear, scalar relativistic scheme, without the spin-orbit coupling taken into account. In the both cases, the band splitting is absent in the results. Please note the difference in the first Brillouin zone for the normal and the distorted phase of 1T-TaSSe—the relation of both zones is illustrated in Figure A1c.
